# Genome sequences of lower Great Lakes *Microcystis* sp. reveal strain-specific genes that are present and expressed in western Lake Erie blooms

**DOI:** 10.1371/journal.pone.0183859

**Published:** 2017-10-11

**Authors:** Kevin Anthony Meyer, Timothy W. Davis, Susan B. Watson, Vincent J. Denef, Michelle A. Berry, Gregory J. Dick

**Affiliations:** 1 Cooperative Institute for Great Lakes Research (CIGLR), University of Michigan, Ann Arbor, MI, United States of America; 2 Department of Earth and Environmental Sciences, University of Michigan, Ann Arbor, MI, United States of America; 3 NOAA Great Lakes Environmental Research Laboratory, Ann Arbor, MI, United States of America; 4 Environment and Climate Change Canada, Burlington, ON, Canada; 5 Department of Ecology and Evolutionary Biology, University of Michigan, Ann Arbor, MI, United States of America; INRA, FRANCE

## Abstract

Blooms of the potentially toxic cyanobacterium *Microcystis* are increasing worldwide. In the Laurentian Great Lakes they pose major socioeconomic, ecological, and human health threats, particularly in western Lake Erie. However, the interpretation of “omics” data is constrained by the highly variable genome of *Microcystis* and the small number of reference genome sequences from strains isolated from the Great Lakes. To address this, we sequenced two *Microcystis* isolates from Lake Erie (*Microcystis aeruginosa* LE3 and *M*. *wesenbergii* LE013-01) and one from upstream Lake St. Clair (*M*. *cf aeruginosa* LSC13-02), and compared these data to the genomes of seventeen *Microcystis* spp. from across the globe as well as one metagenome and seven metatranscriptomes from a 2014 Lake Erie *Microcystis* bloom. For the publically available strains analyzed, the core genome is ~1900 genes, representing ~11% of total genes in the pan-genome and ~45% of each strain’s genome. The flexible genome content was related to *Microcystis* subclades defined by phylogenetic analysis of both housekeeping genes and total core genes. To our knowledge this is the first evidence that the flexible genome is linked to the core genome of the *Microcystis* species complex. The majority of strain-specific genes were present and expressed in bloom communities in Lake Erie. Roughly 8% of these genes from the lower Great Lakes are involved in genome plasticity (rapid gain, loss, or rearrangement of genes) and resistance to foreign genetic elements (such as CRISPR-Cas systems). Intriguingly, strain-specific genes from *Microcystis* cultured from around the world were also present and expressed in the Lake Erie blooms, suggesting that the *Microcystis* pangenome is truly global. The presence and expression of flexible genes, including strain-specific genes, suggests that strain-level genomic diversity may be important in maintaining *Microcystis* abundance during bloom events.

## Introduction

Genetic and physiological differentiation can result in the emergence of closely related populations that are ecologically distinct [1, 2]. This differentiation is encoded in part by “flexible” genes that are frequently gained and lost [3, 4]. In instances where this divergence yields differences in niche occupation the populations represent different ecotypes [5]. Ecotypes have been identified and tracked in populations over seasonal cycles [4] and multiple years [6], and across environmental [7–9] and geographic gradients [10–12]. Some of the best-documented studies of bacterial ecotypes were done on the abundant and widespread cyanobacteria *Prochlorococcus* and *Synechococcus*, for which ecotypes have been defined that have distinct adaptations to light, nutrient availability, and phage resistance [7, 8, 13].

Whereas cyanobacteria such as *Prochlorococcus* and *Synechococcus* are globally important primary producers [14, 15], some cyanobacteria have an innate ability to form large, harmful, and sometimes toxic blooms that impact ecosystem dynamics and threaten freshwater ecosystems, regional economies, recreational activities, and drinking water supplies [16–19]. Among these harmful cyanobacteria, *Microcystis* is one of the most widely reported, described from large blooms in lakes, ponds, reservoirs, and rivers of every continent except Antarctica [18, 20, 21]. However, factors contributing to the worldwide success of *Microcystis* are largely uncharacterized and there remains considerable variance in its response to management efforts [22]. Based on average nucleotide identity, 16S rRNA sequence homology, and DNA-DNA hybridization, all known strains of *Microcystis* belong to the same species complex [21]. However, unlike *Prochlorococcus* and *Synechococcus*, no patterns have been observed in the biogeographic distribution of *Microcystis* genotypes. Genetic variation between strains from the same continent can be higher than between continents [23] and some strains have a large geographic distribution [23, 24].

The dynamic genotypic and phenotypic nature of M*icrocystis* is evident in the high abundance of insertion sequences, transposable elements, restriction enzymes, and genes likely acquired through lateral transfer [25, 26]. This includes the phylogenetic distribution of *mcyA-J* genes encoding biosynthesis of microcystins, the most commonly detected cyanotoxins globally [21, 27, 28]. Phylogenetic analysis of both individual genes and multiple loci within that gene cluster have found toxicity to be polyphyletic with clusters of toxic strains, non-toxic strains, and a combination of both [29–31]. Thus, toxicity appears to be a genetic element that is stable only in the short-term [30]. Indeed, *Microcystis* has lost the ability to produce microcystins in multiple instances over evolutionary time, indicating that toxicity was not essential, or potentially too costly, for survival in particular ecosystems or under certain environmental conditions [32].

Despite the high genetic variability between strains and apparent lack of biogeography, subclades of strains based on phylogenetic relationships of core genes are well preserved, suggesting some degree of phylogenetic cohesion [30, 31]. Further, individual genes display biogeographical patterns [33], and hydrologically linked systems show genetic connectivity in toxic strains of *Microcystis* through the *mcyA* gene [34]. These conflicting patterns likely result from a balance between genome plasticity (rapid gain, loss, or rearrangement of genes), which generates potentially useful variation [35], and genome stability (restriction of gene transfer and maintenance of core genes), which preserves useful variants and resists harmful elements [36].

One hypothesis developed by previous studies is that the conserved subclades of *Microcystis* represent ecotypes (physiologically different strains) [26, 37] or cryptic ecotypes (a phylogenetic cluster that is new or low frequency but ecologically distinct) [30, 38]. In this case, the high genetic diversity observed in co-occurring *Microcystis* strains may represent distinct ecotypes that provide the genetic variation needed for ecological divergence through selection processes [30]. An alternative hypothesis is that stable niches are a prerequisite for ecotype differentiation, and that potential niches available to *Microcystis* are insufficiently stable. As a result, genome plasticity represents an efficient strategy for dynamic environments [39] by rapidly generating variants that may be adapted to new environmental conditions [31, 40]. The genome plasticity of *Microcystis* may therefore have been selected for through both gene content and transcription [33, 40], creating phenotypic variation within a localized population or bloom adapted for specific environmental conditions [38].

The Laurentian Great Lakes have a history of large-scale cyanobacterial blooms dominated by *Microcystis*, with the largest blooms typically occurring in western Lake Erie [22, 41–44]. Currently, only one genome of cultured *Microcystis* from the Laurentian Great Lakes (Lake Michigan) has been described [31]. This represents a critical gap for genomic and metagenomic studies that rely on mapping sequence reads to available genomes from reference strains [21, 45–47]. Our goal was to close this knowledge gap by sequencing the genomes of three lower Great Lakes (LGL) *Microcystis* strains, using comparative genomics to place them in the framework of other *Microcystis* genomes. Further, analysis of the content and expression of their strain-specific genes at several stages of the 2014 Lake Erie bloom reveals how genetic variation manifests in natural communities across changing environmental conditions, providing insights into whether differences in gene content and expression of LGL *Microcystis* strains could represent ecotypes and how these differences contribute to bloom formation, proliferation, and toxicity.

## Methods

### *Microcystis* strain culture and DNA extraction

Whole genome analysis of 20 *Microcystis* strains was conducted (Table 1). Of these genomes, three are newly sequenced isolates from the LGL (*Microcystis cf aeruginosa* LSC13-02, *Microcystis aeruginosa* LE3, and *Microcystis wesenbergii* LE013-01) while the other 17 have been previously compared and were obtained from the Joint Genome Institute Integrated Microbial Genomes & Microbiomes (JGI IMG/M) databases [21, 31, 36, 48–54]. Isolates LE3 and LE013-01 were isolated from western Lake Erie [43], and LSC13-02 was isolated from Lake St. Clair, which is both physically and genetically connected to Lake Erie via the Detroit River [34].

**Table 1:** Comparative genomic features of 17 publicly available *Microcystis* genomes (Downloaded from JGI IMG/M) [52] and three strains isolated from the lower Great Lakes (in bold).

**Strain**	**Culture Reference**	**Isolation date**	**Genome Size (Mbp)**	***mcy* genes?**	**Strain-specific genes**	**% core genes**	**Strain-specific % present**	**Strain-specific % expressed**
*Microcystis aeruginosa*	PCC 9807	1973	5.1	Yes	594	36.9	98.0	85.5
*Microcystis aeruginosa*	PCC 9443	1954	5.1	Yes	683	36.7	97.2	81.1
*Microcystis aeruginosa*	PCC 9432	Sep.1954	5.0	No	316	38.2	98.7	78.5
*Microcystis aeruginosa*	PCC 7005	1946	4.9	No	561	37.0	97.2	82.0
*Microcystis aeruginosa*	PCC 9808	1973	5.0	Yes	448	37.4	98.0	78.6
*Microcystis aeruginosa*	PCC 7941	Sep.1954	4.8	Yes	226	40.7	96.9	81.9
*Microcystis aeruginosa*	**LE3**	**1996**	**4.6**	**Yes**	**189**	**43.0**	**99.5**	**88.9**
*Microcystis aeruginosa*	TAIHU98	1997	4.9	No	331	39.8	99.1	89.7
*Microcystis aeruginosa*	SPC 777	Feb.2000	5.5	Yes	471	38.8	92.6	86.6
*Microcystis aeruginosa*	PCC 7806	1972	5.3	Yes	392	37.1	99.5	90.3
*Microcystis aeruginosa*	DIANCHI905	1998	4.9	Yes	283	39.7	97.2	81.6
*Microcystis aeruginosa*	PCC 9806	Aug.1975	4.2	No	398	45.5	98.2	84.7
*Microcystis sp*.	T1-4	Unknown	4.7	No	525	40.9	99.2	80.4
*Microcystis aeruginosa*	PCC 9701	1996	4.7	No	442	40.3	98.1	88.2
*Microcystis wesenbergii*	**LE013-01**	**Aug.2013**	**4.0**	**No**	**222**	**48.6**	**100**	**83.8**
*Microcystis aeruginosa*	NIES-44	Aug.1974	4.6	No	292	43.3	94.9	87.3
***Microcystis cf aeruginosa***	**LSC13-02**	**Aug.2013**	**4.3**	**Yes**	**458**	**44.9**	**99.8**	**89.1**
*Microcystis aeruginosa*	PCC 9717	1996	5.2	Yes	642	36.0	97.5	96.1
*Microcystis aeruginosa*	PCC 9809	Aug.1982	4.9	Yes	526	37.4	98.1	85.6
*Microcystis aeruginosa*	NIES-843	Aug.1997	5.8	Yes	526	33.7	99.4	89.9

**Fig 1:**
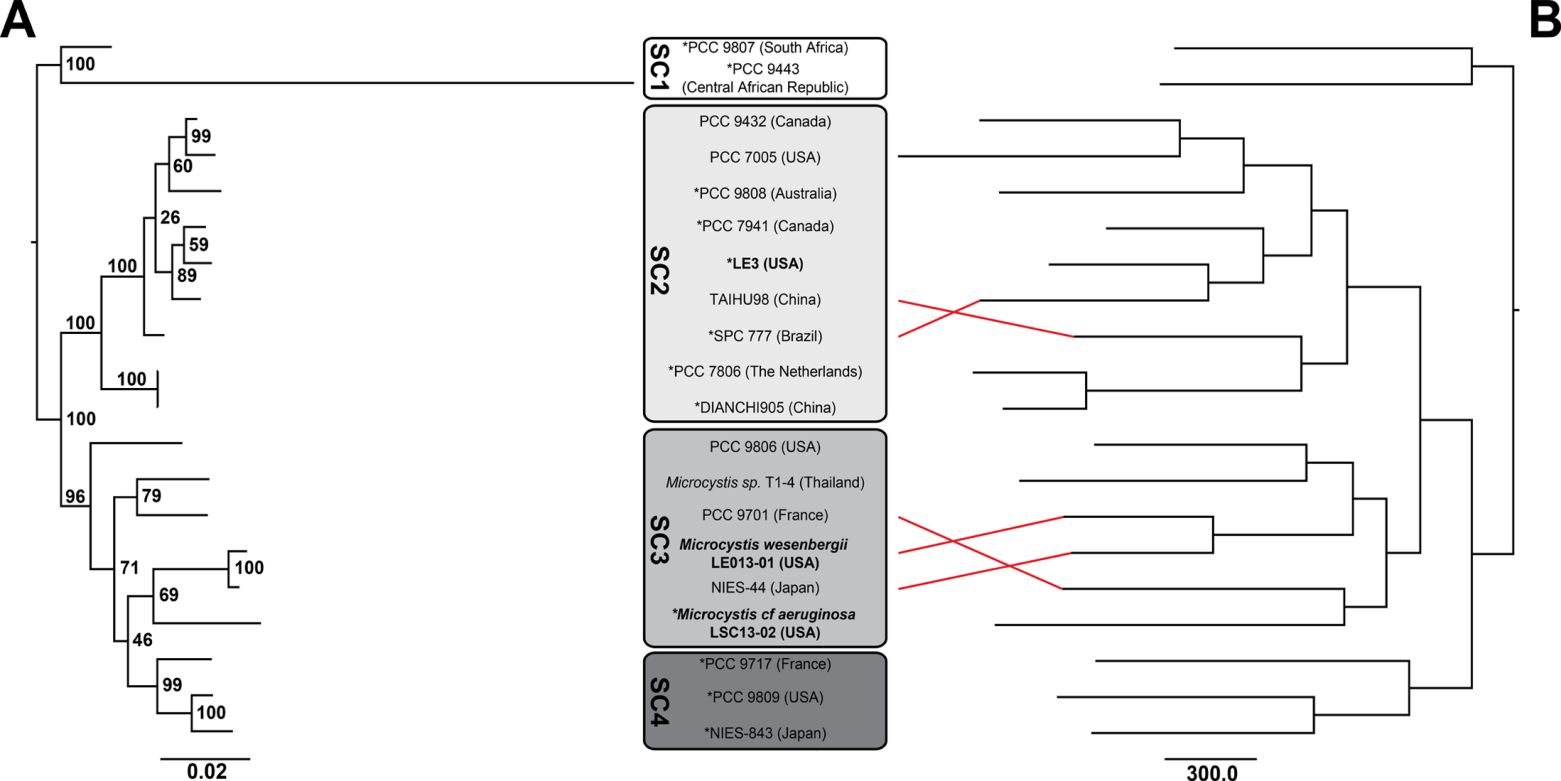
Cladograms of twenty *Microcystis* strains based on A- phylogenetic analysis using RAxML inference on concatenated alignments of six housekeeping genes (*ftsZ*, *glnA*, *gltX*, *pgi*, *recA*, and *tpi*), ribosomal protein S3, global nitrogen regulator (*ntcA*), and phycocyanin subunit B (*cpcB*) genes and B- parsimony search of presence/absence of flexible genes within each genome. SC = Subclade as assigned by Humbert et al. (2013) [31], red lines indicate where phylogenetic assignments differ between trees A and B, *strains which are toxic based on the presence of the *mcy* gene operon.

*Microcystis aeruginosa* strain LE3 and *Microcystis wesenbergii* strain LE013-01 were isolated from Lake Erie in the summer of 1996 and August, 2013, respectively. *Microcystis cf aeruginosa* strain LSC13-02 was isolated from Lake St. Clair in August, 2013. Strain–specific % present and expressed columns represent the proportion of strain-specific genes found in the metagenome and metatranscriptomes from a 2014 Lake Erie *Microcystis* bloom. Horizontal bars indicate subclades as assigned by Humbert et al. (2013) [31] and as presented in Fig 1.

Individual isolates (15mL) were grown in BG-11 medium [55] in an incubation room (20°C, 38 μE m^-2^ s^-2^, 12h Light:Dark cycle). Cells were spun down at ~9,400 × g for 10 minutes in a benchtop centrifuge, the supernatant was decanted and the pelleted cellular material was frozen at -80°C until subsequent DNA extraction, purification, and sequencing. Frozen cell pellets were thawed at room temperature and then extracted using a Qiagen DNeasy® Blood and Tissue Kit (Qiagen, Hilden, Germany). Briefly, samples were incubated with 100μL Qiagen ATL tissue lysis buffer, 300μL Qiagen AL lysis buffer, and 30μL proteinase K at 56°C for 1 hour with agitation, followed by mixing with a vortexer at maximum speed for 10 minutes. Lysates were homogenized with a QiaShredder™ spin-column before purification according to the DNeasy® protocol.

### Lake Erie sampling and RNA extractions

Field samples were collected in conjunction with the joint NOAA Great Lakes Environmental Research Laboratory / University of Michigan Cooperative Institute for Great Lakes Research weekly sampling program for western Lake Erie. In 2014, six sites were sampled bi-monthly in June then weekly from July through October. From this sampling effort we focused on representative samples to capture each stage of the *Microcystis* bloom; early bloom (late July-early August), mid-bloom (late August), and late/post-peak bloom (September-October). Bloom stages were determined by phycocyanin fluorescence and relative abundance [56]. Samples were chosen from three of the regularly sampled stations (WE2, near to the mouth of Maumee River, 41° 45.743’ N, 83°19.874’ W; WE4, offshore towards the center of the western basin, 41°49.595’ N, 83°11.698’ W; and WE12, adjacent to the water intake crib for the city of Toledo, 41°42.535’ N, 83°14.989’ W) [56]. Metagenomic data were generated from samples collected from station WE12 in early blooms stages, and metatranscriptomic data were generated from early (1 sample each from all three stations, WE2 21.July.2014, WE4 29.July.2014, and WE12 4.August.2014), middle (1 sample from station WE12 only, 25.August.2014), and late (1 sample each from all three stations, WE2 6.October.2014, WE4 8.September.2014, WE12 23.September.2014) bloom stage samples. All samples were collected shortly after arriving on-station using a peristaltic pump to obtain 2L of water integrated from 0.1m below the surface to 1m above the bottom [56]. This was then filtered onto 100μm polycarbonate 47mm filter in a Swinnex™ filter holder using a sterile 60mL syringe. A pore size of 100 μm was used to maximize the amount of *Microcystis* colonies retained on the filter while excluding smaller particles. Previous work has shown that in Lake Erie the > 100 μm *Microcystis* community comprised over 90% of all *Microcystis* cells in the water column [57, 58]. Filters were then immersed in 1 mL RNALater™ (Invitrogen™ Ambion™) and placed on ice during transport before being stored at -80°C. RNA was extracted from samples using RNeasy Mini Kit (QIAGEN) according to manufacturer’s instructions.

### Assembly and binning

Shotgun sequencing of DNA and RNA was performed on the Illumina® HiSeq™ platform (2000 PE 100, Illumina, Inc., San Diego, CA, USA) at the University of Michigan DNA Sequencing Core. Sequence reads were put through a quality control pipeline that consisted of two runs of FASTQC version 0.10.0 (http://www.bioinformatics.babraham.ac.uk/projects/fastqc/), dereplication (100% identity over 100% of length), adapter removal using Scythe [59], and read trimming using Sickle [60].

The genome of each *Microcystis* isolate was assembled *de novo*. We used the iterative de Bruijn graph approach for uneven sequencing depths (IDBA-UD) [61] for all assemblies with the following parameters: minimum kmer size 52, maximum kmer size 92, step size 8, minimum contig 500. Read coverage was generated by mapping paired end reads to the assembled contigs using the Burrows-Wheeler Aligner (BWA version 0.7.9a-r786) [62] with default parameters. Paired forward and reverse alignments were generated in SAM format and read counts extracted using SAMtools 1.0 [63]. Because the *Microcystis* cultures were not axenic, assembled contigs were binned into putative taxonomic groups with emergent self-organizing maps (ESOM) of tetranucleotide frequencies (Robust ZT transformation) using Databionics ESOM Tools [64] (http://databionic-esom.sourceforge.net). Only contigs longer than 4kb were considered for ESOM binning, and longer contigs were chopped into 10kb windows for this analysis. The other ESOM parameters were as follows: training with a K-Batch algorithm (k = 0.15%) for 40 training epochs, a standard best match search method with a local best match search radius of 8, a Gaussian weight initialization, Euclidean data space function, a starting training radius of 204 with linear cooling to 1, and a starting learning rate of 0.5 with linear cooling to 0.1. Bin taxonomy was identified using a combination of: (i) BLASTN of contigs [65] against the Silva SSU Database version 119 [66, 67]; (ii) ESOM binning with *Microcystis aeruginosa* DIANCHI905 and PCC 9808 as reference genomes; (iii) phylogenetic analysis using the full marker set in the PhyloSift pipeline [68].

The three newly sequenced *Microcystis* genomes were individually submitted to the Integrated Microbial Genomes database (US DOE JGI/IMG) for gene calling [52–54]. All newly sequenced genome files are available from the Integrated Microbial Genomes Database (US DOE JGI/IMG: Taxon IDs 2606217223, 2606217222, and 2606217221) and NCBI (Genome accession numbers MTBS00000000, MTBU00000000, and MTBT00000000; Sequence Read Archive accession numbers SRR5144737, SRR5145065, and SRR5145066; BioProjects PRJNA340013, PRJNA340089, and PRJNA340134; BioSamples SAMN05629279, SAMN05645801, and SAMN05645814). Gene annotations are available from JGI/IMG (analysis project IDs GA0066243, GA0066240, and GA0066226).

### Phylogenetic analysis

Nucleic acid sequences for six housekeeping genes (Cell division protein FtsZ, glutamine synthetase, Glutamyl-tRNA Synthetase, glucose-6-phosphate isomerase, DNA repair protein recA, and triose phosphate isomerase) [31], as well as the ribosomal protein S3, global nitrogen regulator (*ntcA*), and phycocyanin subunit B gene (*cpcB*) were obtained from the NCBI Nucleotide database or IMG for all 20 *Microcystis* genomes. Sequences were aligned using the MUSCLE tool [69] in MEGA6 (Version 6.06; Build 6140226) [70] and concatenated using ARB v5.5 [71]. Phylogenetic inferences were made on concatenated alignments with Randomized axelerated maximum likelihood (RAxML) using RAxML-VI-HPC v8.1.15 with a total of 1000 iterations [72]. A second, maximum parsimony tree was created through GET_HOMOLOGUES with the PARS program of the PHYLIP suite [73] using presence/absence data of the flexible genome compiled into a pan-genome matrix from OMCL clusters of only the flexible genes [74]. Phylogenetic trees were formatted using FigTree v1.4.2 [http://tree.bio.ed.ac.uk] to identify previously established *Microcystis* sp. subclades [31] and joined using Adobe® Illustrator® CS6.

### Core and pan-genome analysis

In order to standardize the gene calling and annotation for the purpose of comparative genomics, we used the Prokaryotic dynamic programming gene-finding algorithm (Prodigal) to predict genes in all 20 *Microcystis* genomes [75]. Prodigal was first run in training mode to establish *Microcystis aeruginosa* NIES-843 (the only complete *Microcystis* genome used in this study) [25] as a reference genome followed by a run in normal mode with all 20 genomes. Annotations were then re-assigned by a BLASTN of annotated genome sequences obtained from IMG or NCBI to the gene sequences called by Prodigal, keeping only the BLAST hits with a minimum bitscore of 100 and percent identify match of 95%.

Comparative genomics analysis was done using the GET_HOMOLOGUES software package [74]. Orthologous gene families were identified using the OrthoMCL clustering algorithm (OMCL) with a sequence cluster reporting value of t = 0 and no Pfam-domain composition requirements [74, 76, 77]. This approach uses the exponential decay models of Tettelin [78] and Willenbrock [79] to calculate the core-genome size and the exponential model of Tettelin to estimate the pan-genome size (the sum of both genes shared amongst all strains and genes strain-specific to individual strains) [78]. Strain-specific genes, genes found in only a single *Microcystis* isolate, were identified using the *parse_pangenome_matrix*.*pl* script with the pan-genome set of each individual strain being compared to those of all other genomes [76].

### Metagenomic and metatranscriptomic analysis

Raw reads from the 2014 bloom metagenomic and metatranscriptomic data were mapped to the strain-specific genes identified in GET_HOMOLOGUES, called genes from IMG, and the housekeeping, ribosomal protein S3, *ntcA*, and *cpcB* genes used in the phylogenetic analysis using the BWA mapper with default parameters [62]. Strain-specific gene reads were then normalized by the average read coverage of the genes used in the phylogenetic analysis. Annotation data for strain-specific genes were obtained from IMG and the proportion of genes in each COG was calculated using the normalized read coverage.

## Results

### Features and phylogeny of three new draft genomes from lower Great Lakes *Microcystis* strains

Basic features of the three new draft genomes of *Microcystis* strains from the lower Great Lakes (LGL) are shown in Table 1. Approximately 70% of the coding DNA sequences in the LGL genomes had functional annotations. Comparative genomics showed that every *Microcystis* strain had strain-specific genes (not found in any other *Microcystis* strain used in this study). The number of strain-specific genes for the LGL strains of *Microcystis* was 189, 222, and 458, in LE3, LE013-01, and LSC13-02, respectively. The most abundant functional categories for completely strain-specific genes were tranposases (2.9%), transferases (5.9%), and endonucleases (2.6%).

Two methods were used to evaluate the genetic relationships of sequenced *Microcystis* strains: maximum likelihood analysis of nine core genes and maximum parsimony based on the content of flexible genes within each genome (Fig 1). There was good overall congruence between the trees produced by these two methods. Only the placement of two pairs of strains differed, *Microcystis aeruginosa* SPC 777 and TAIHU98, and PCC 9701 and PCC 9806, and both of these differences were within subclades defined previously [31].

Based on 20 genomes of *Microcystis* the core-genome was estimated to be 2008 and 1924 genes with residual standard errors of 174 and 134 for the Tettelin and Willenbrock models, respectively. The pan-genome for these 20 *Microcystis* strains was estimated to be 8620 genes with a residual standard error of 196. Within any given *Microcystis* strain, the genome consisted of 34–49% core genes and 51–66% flexible genes.

### Occurrence of foreign genetic defense systems in lower Great Lakes isolates of *Microcystis*

**Fig 2:**
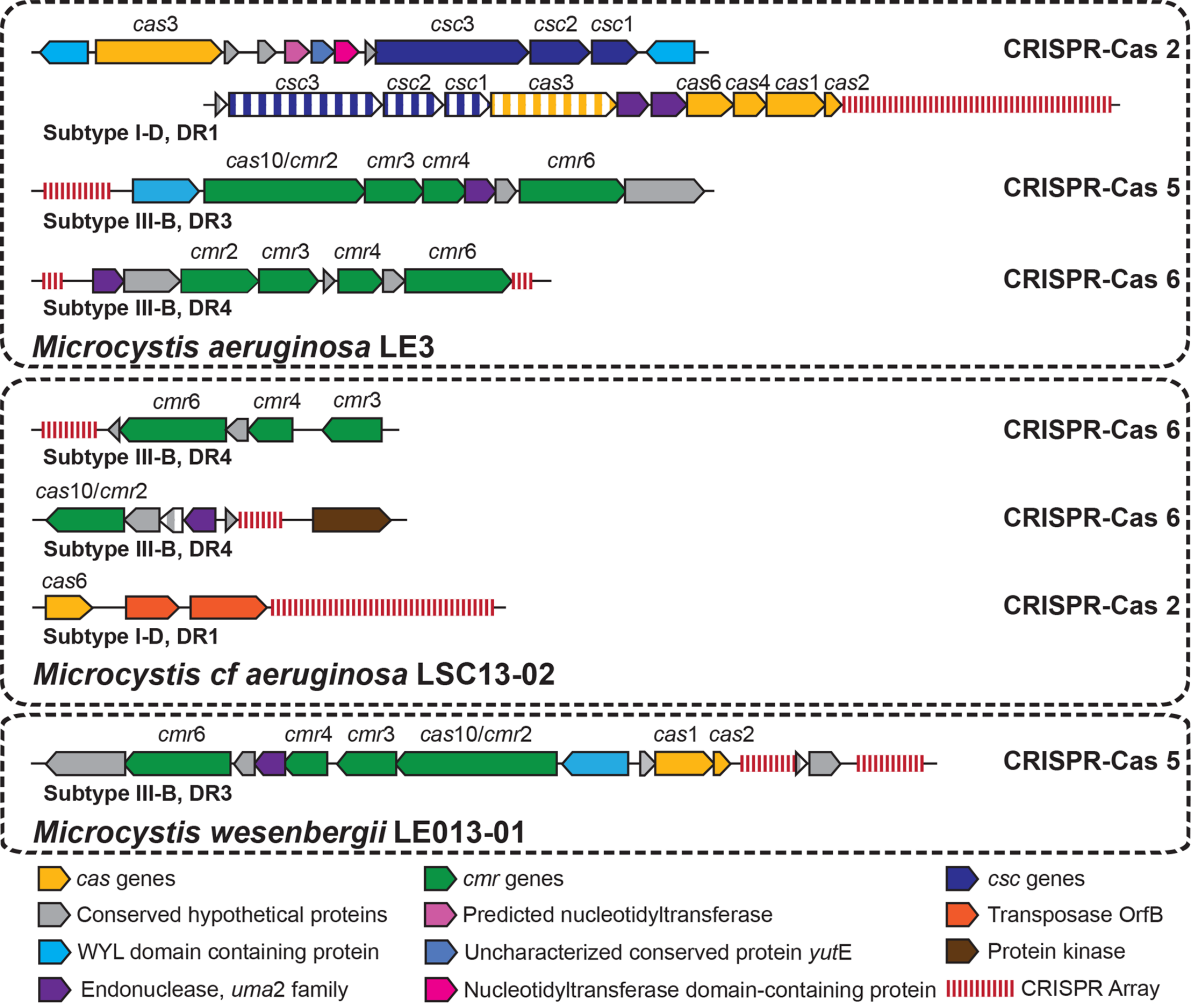
Organization and repeat structure of CRISPR-Cas arrays identified in lower Great Lakes *Microcystis* strains. Genes colored with vertical stripes indicate genes strain-specific to that particular strain of *Microcystis*. CRISPR-Cas categories, subtypes, and repeat types were based on architecture described by Yang et al. (2015) [36].

Multiple strain-specific genes associated with genomic plasticity were found in LGL *Microcystis* isolates, several of which defend against foreign genetic elements (Table 2). Strain LE3 had four strain-specific genes identified as *csc* and *cas* components of the CRISPR-Cas system (Fig 2), which defends against invading genetic elements [80]. The CRISPR-Cas system found in strain LE3 has an architecture and array repeat similar to that of the *Microcystis* Subtype I-D CRISPR-Cas 2 and DR1 [36]. The LE3 CRISPR-Cas 2 differed from those in 11 of the 19 other *Microcystis* genomes (NIES-843, PCC 9443, PCC 7806, PCC 9808, PCC 7941, PCC 9717, PCC 9809, PCC 9807, DIANCHI905, PCC 9432, and PCC 9701) in that *cas*3 was not adjacent to the transcriptional regulator but was instead between *csc*1 and *uma*2 family endonucleases adjacent to the *cas*6 gene. There were also second copies of *csc*1, *csc*2, *csc*3, and *cas*3 near the beginning of the scaffold. Subtype III-B CRISPR-Cas 5 and Subtype III-B CRISPR-Cas 6 (with DR3 and DR4 repeat arrays) were also found, but with none of the associated genes being classified as strain-specific. Both CRISPR-Cas 5 and CRISPR-Cas 6 were most similar to that found in *Microcystis* TAIHU98. In addition there was a polyphosphate kinase (*ppk*) gene located upstream of two CRISPR arrays (DR2 repeat type) with 3 hypothetical proteins and an mRNA interferase (RelE/StbE) between them, which could be the remnants of a Subtype III-B CRISPR-Cas 4 system. Strain LE3 also had 81 restriction enzymes and 6 DNA restriction-modification genes, of which 3 restriction endonucleases were strain-specific.

**Table 2:** Genes associated with genomic plasticity and resistance to foreign genetic elements in lower Great Lakes *Microcystis* strains.

Strain	CRISPR-Cas systems	Restriction-modification	Strain-specific restriction-modification	Transposases	Strain-specific transposases
**LE3**	3	87	3	46	5
**LE013-01**	1	86	5	47	5
**LSC13-02**	2	95	20	45	13

Strain LSC13-02 contained two versions of Subtype III-B CRISPR-Cas 6, both with the DR4 repeat [36]. One had an organization most similar to that of PCC 9807 and the second had an organization similar to that of *Microcystis* PCC 7941, PCC 7806, and DIANCHI905. The second CRISPR-Cas 6 array differed from previously described arrays with a protein kinase oriented away from the CRISPR array (as opposed to a polyphosphate kinase running towards the CRISPR array) and a strain-specific hypothetical protein between the CRISPR array and *cas10/cmr2*. There is also a large CRISPR array that is downstream of *Cas6* with two transposases between the *Cas6* protein and the CRISPR array that could be a partial Subtype I-D CRISPR-Cas 2 given that it has a DR1 repeat [36]. Strain LSC13-02 also had 91 restriction enzymes and 4 restriction-modification proteins. 20 of these were strain-specific to strain LSC13-02.

Strain LE013-01 possessed Subtype III-B CRISPR-Cas 5 with the same organization and repeat type (DR3) as four other strains of *Microcystis* (PCC 9701, PCC 7941, DIANCHI905, and PCC 9432) with a strain-specific hypothetical protein between that CRISPR array and a second array. LE013-01 also had 83 restriction enzymes and 3 restriction-modification proteins, 5 of which were strain-specific.

### Occurrence and expression of strain-specific genes in western Lake Erie

One metagenomic and five metatranscriptomic data sets from western Lake Erie were mapped against strain-specific gene sequences for 20 *Microcystis* strains to determine the presence and expression of those genes in the environment under different bloom conditions. These bloom conditions do not necessarily represent natural cell growth cycles but are rather operationally defined by trends in biomass and pigment through the season on a basin-wide scale. Over 92% of strain-specific genes from each strain studied here were present in Lake Erie metagenomic data (Table 1). Of these genes found in the LGL strain metagenomes, 168 of 189 LE3, 186 of 222 LE013-01, and 408 of 458 LSC13-02 genes were also present in the metatranscriptomic data of at least one of the three bloom stages. There was no clear enrichment of strain-specific genes from the LGL strains (LE3, LE013-01, and LSC13-02) relative to other *Microcystis* strains from around the world in the Lake Erie metagenome or metatranscriptome.

**Fig 3:**
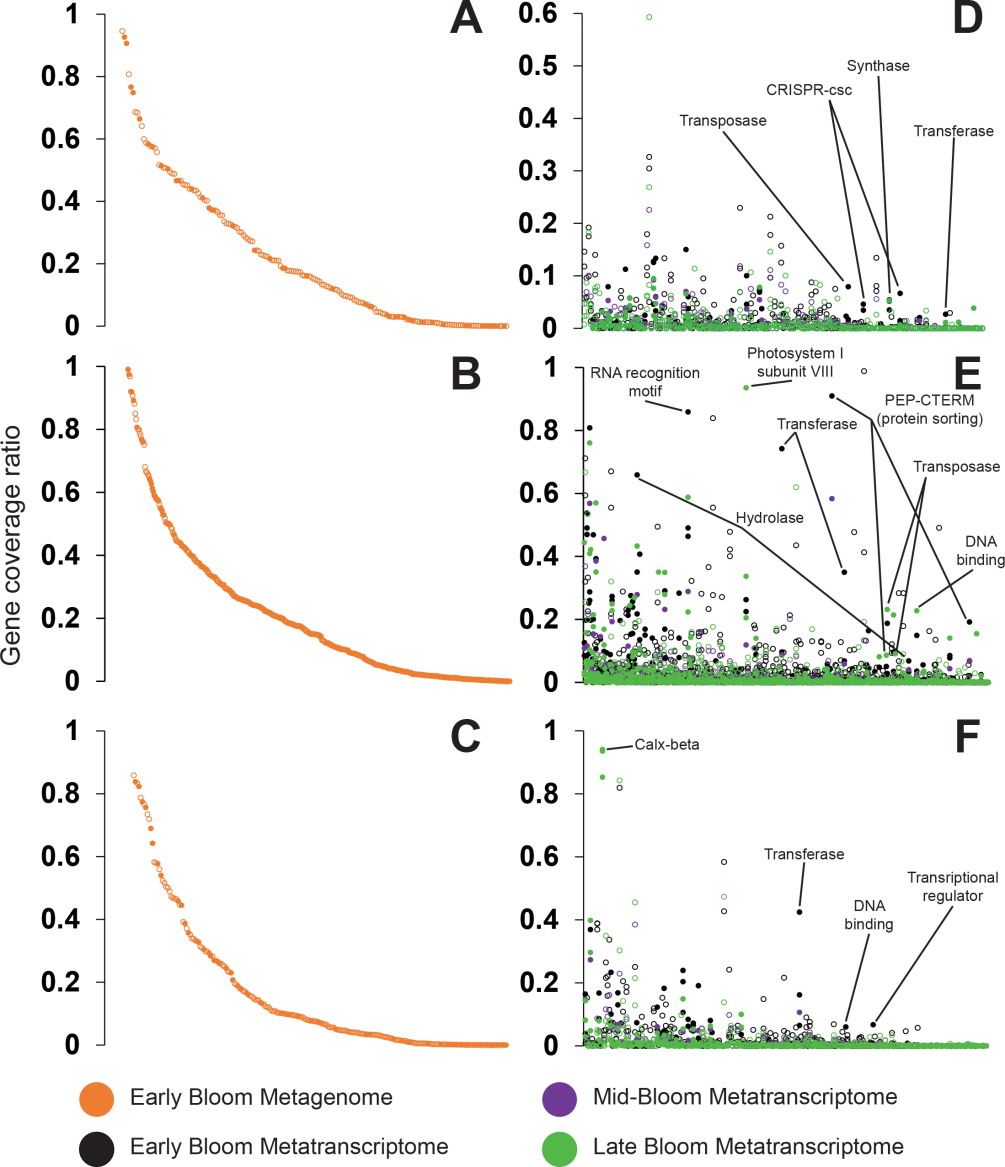
Rank abundance of the coverage of strain-specific genes from LGL *Microcystis* isolates in metagenomic and metatranscriptomic data from multiple bloom stages of the 2014 Lake Erie *Microcystis* bloom. Coverage is presented as the ratio of strain-specific genes compared to average coverage for marker genes used in the phylogenetic analysis. (A, B, C) Metagenomic data, (D, E, F) Metatranscriptomic data with genes ranked by metagenomic abundance. (A, D) strain LE3; (B, E) strain LSC13-02; (C, F) strain LE013-01. Closed circles have an annotated function, open circles are hypothetical proteins.

To evaluate the relative abundance of these strain-specific genes and their transcripts in Lake Erie *Microcystis* cells, we next compared their coverage in the metagenomic and metatranscriptomic data to that of phylogenetic marker genes. Because these marker genes are present in the *Microcystis* genomes in a single copy, for the metagenome this ratio provides an estimate of the fraction of cells containing the strain-specific genes. For the metatranscriptome the ratio provides a qualitative estimate of expression of the strain-specific genes relative to housekeeping genes. We focused on samples in which *Microcystis* was abundant (based on phycocyanin and 16S rRNA gene data [56]), including the metagenomic data from the early bloom and metatranscriptomic data from the early, mid, and late blooms. The frequency of strain-specific genes varied widely in the 2014 *Microcystis* bloom. Approximately 16% of strain-specific genes were present at > 50% of the relative abundance of phylogenetic marker genes, suggesting they are present in the majority of *Microcystis* cells in Lake Erie. The remaining 84% of strain-specific genes are present at < 50% of phylogenetic marker gene relative abundance, indicating that they are more rare in the Lake Erie *Microcystis* populations (Fig 3).

Expression patterns of strain-specific genes varied across western Lake Erie and throughout the bloom event. Most strain-specific genes were expressed at levels < 50% of the average of marker genes or not at all. At station WE12 (Toledo water intake crib), the number of strain specific genes expressed at any level decreased from 72% in the early bloom to 58% in the late bloom. In contrast, stations WE2 (Maumee River outflow) and WE4 (offshore, towards central Lake Erie) showed an opposite pattern. At station WE2 31% of strain-specific genes were expressed in the early bloom and 58% in the late bloom and at Station WE4 50% and 67% of strain-specific genes were expressed in the early and late blooms, respectively.

**Fig 4:**
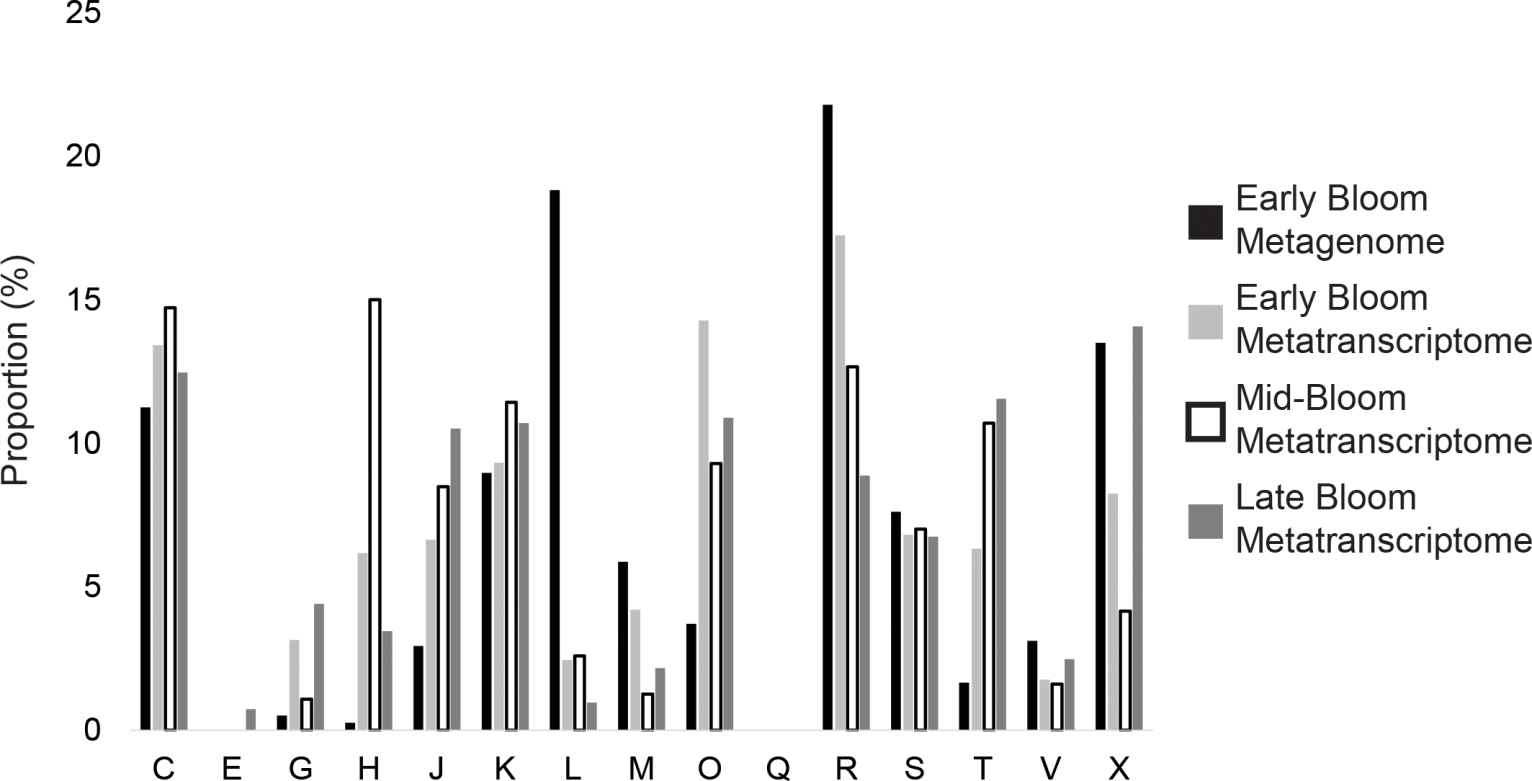
Relative abundance of strain-specific genes in (A) metagenomes and (B) metatranscriptomes from multiple bloom stages of the 2014 Lake Erie *Microcystis* bloom, annotated by COG category. Relative abundance was determined by normalizing the metagenomic and metatranscriptomic strain-specific gene read coverage by the read coverage of phylogenetic marker genes. COG categories are: C- Energy production and conversion, E- Amino acid transport and metabolism, G- Carbohydrate transport and metabolism, H- Coenzyme transport and metabolism, J- Translation, ribosomal structure, and biogenesis, K- Transcription, L- Replication, recombination, and repair, M- Cell wall/membrane/envelope biogenesis, O- Posttranslational modification, protein turnover, chaperones, Q- Secondary metabolites biosynthesis, transport, and catabolism, R- General function prediction only, S- Function unknown, T- Signal transduction mechanisms, V- Defense mechanisms, X- Mobilome: prophages, transposons.

We next analyzed the functional profile of the strain-specific genes in the Lake Erie metagenome and metatranscriptomes. Strain-specific genes in the metagenomic data were dominated by functional categories related to (i) “general function”, (ii) replication, recombination and repair; (iii) mobilome; prophages, transposons, and (iv) energy production and conversion (Fig 4). The relative abundance of functional categories was variable across bloom stages in the metatranscriptomic data (Fig 4). Of the strain-specific genes expressed in the metatranscriptomic data with a functional annotation, many were associated with genomic plasticity including transposases, endonucleases, transcriptases, integrases, and CRISPR-Cas proteins. Transposases included two different IS4 family transposases and a DDE domain transpose at the start of a large operon for pigment synthesis in strain LE3, DDE domain transposases in strain LE013-01, and an IS605 OrfB family transposase in strain LSC13-02. Transcripts mapped to strain-specific restriction endonucleases in strains LE3 and LSC13-02 and DNA binding helix-turn-helix endonucleases in all three LGL strains. Transcripts related to foreign genetic elements included Subtype I-D CRISPR-Cas 2 proteins in strain LE3, a reverse transcriptase and a phase integrase in strain LSC13-02, and a plasmid stabilization ParE toxin of the ParDE toxin-antitoxin system in strain LE013-01.

The next most abundant type of annotated strain-specific genes found in the Lake Erie metatranscriptomic data were associated with transcription or metabolism. Methyltransferases were identified in all three LGL strains, matching *ecoRI* adenine-specific methyltransferase in strain LSC13-02, *fkbM* domain methyltransferases in both LE3 and LE013-01, and DNA methyltransferase (*dcm*) in LE3. Transcripts also matched to transcriptional regulators in the *xre* and *snf*2 families, a colonic acid biosynthesis glycosyltransferase, dolichyl-phosphate-mannose protein, and ribosomal S18 acetylase in strain LE013-01. There were also transcripts that mapped to strain-specific genes in strain LE3 identified as 2Fe-2S ferredoxin, endopeptidase, and protein phosphatase 2C, associated with electron transfer, peptide bond cleavage, and Mg^2+^/Mn^2+^ -dependent enzymes involved in stress signaling, respectively.

Several strain-specific genes associated with cell walls and transporters were also found in metatranscriptomic data. Two glycosyltransferases involved in cell wall biosynthesis, identified in strain LE013-01, were found in early bloom stages. Late bloom stages contained a strain-specific N-acetylmuramoyl-L-alanine amidase and a penicillin-insensitive murein endopeptidase, identified in LE3, which cleave links in cell wall peptides. There were also tetratricopeptide repeats (TPR) associated with *xisH* and *xisJ* excision proteins, identified in strain LE3, which are associated with lysogeny in cyanobacteria (given that *Microcystis* is a non-heterocystous cyanobacterium). Transporter related proteins included a cation transporter/ATPase identified in strain LE013-01, a wholly strain-specific scaffold in strain LSC13-02 which contained two genes of the Calx-beta domain of sodium-calcium exchangers, and HEAT repeat domain proteins found in strain LSC13-02, which form cytoplasmic intracellular transporters, that were found in all bloom stage metatranscriptomes. Throughout the bloom there was expression of CARDB proteins, which are cell adhesion related domain proteins, identified from the strain LSC13-02 genome.

## Discussion

We compared genomes of *Microcystis* strains isolated from the lower Great Lakes (LGL) to those from strains that were isolated across the globe and to genes and transcripts from communities of mixed strains in blooms of Lake Erie. This improves the molecular understanding of *Microcystis* by demonstrating the diversity and characteristics of the flexible genome of *Microcystis* and the presence and expression of a majority of the flexible genome during a naturally occurring bloom. We identified genes specific to individual strains, which may confer adaptive or functional advantages that play a role in intraspecific competition [33], adaptation in a changing environment [31, 40, 81], and the composition and succession of blooms [82].

Our comparative analysis of 20 *Microcystis* genomes was consistent with results from an earlier study that identified core and pan-genomes of twelve *Microcystis* genomes [31]. Our findings confirm that the core genome of *Microcystis* is both known and described [30, 31] and indicate that the worldwide genomic diversity of *Microcystis* likely exceeds our capability to sequence and identify previously unknown flexible genes [42, 83].

Based on concatenated alignment of housekeeping genes, our phylogenetic results identified four subclades that are congruent with previous studies that used concatenated alignments of multiple different genes including seven housekeeping genes or all 1,989 core genes [30, 31]. In addition, we found that phylogenies based on the content of the flexible genome are congruent with those from housekeeping and core genes (Fig 1). To our knowledge this is the first evidence that the flexible genome is linked to the core genome of *Microcystis*. Taken together, the absence of a consistent geographic distribution of subclades, the open pan-genome with high potential for gene acquisition [26], genome re-arrangement [32, 42, 84] and loss [33, 85, 86], and the consistent phylogenetic relationships of *Microcystis* core and flexible genes suggest genetic cohesiveness of sub-clades that is independent of geography [21].

To explore the environmental relevance of the strain-specific genes identified here, we assessed their presence in metagenomic and metatranscriptomic data at various stages of a 2014 western Lake Erie *Microcystis* bloom. This included samples of the early bloom that were characterized by elevated toxicity and high environmental nitrate concentration and late stages of the bloom that were characterized by lower toxicity and nitrate concentrations [53, 87]. Intriguingly, the Lake Erie blooms contained strain-specific genes from not only the Great Lakes strains but also from strains isolated from continents across the world. This suggests that the pangenome is truly global, and highlights the value of using the entire pangenome for analyzing and interpreting metagenomic and metatranscriptomic datasets. Because the flexible genome is linked to phylogeny it may be more appropriate to focus on the subclade structure rather than geographic origin when considering and employing reference strains and genomes. The abundance and substantial expression of some of the flexible genes in the environment (Figs 3 and 4) suggests that these genes may be ecologically important, but a key question that remains open is to what extent the flexible genome is linked to phenotype.

Despite strain LE3 having a larger genome than LE013-01 and LSC13-02 it had the fewest strain-specific genes of the three newly sequenced strains. Strain LE3 has been in culture since 1996 while both LE013-01 and LSC13-02 were isolated in 2013. Both physiology and community composition (for non-axenic cultures) can be altered by time in culture [88] and culturing conditions [89, 90] but to our knowledge the impact of time spent in culture on the genomic features or gene composition of cyanobacterial isolates is unknown.

Previous work has shown that the *Microcystis* genome has a large proportion of repeat sequences, transposases, restriction enzymes [26] and insertion sequences [91] compared to other cyanobacteria. This indicates a capacity for *Microcystis* to adapt to different environments through internal genome re-arrangement as well as horizontal gene transfer [39, 40]. These genomic changes can lead to adaptive diversification but may also cause loss of physiological function [40, 83, 91]. A balance is therefore required for *Microcystis* to be able to regulate DNA incorporation and transposition so that essential pathways (core genes) are preserved while allowing for adaptation to environment-specific stressors such as nutrient gradients or phages [40, 47, 87, 92]. Such a combination would ensure genetic stability through the presence and inheritance of genes that are resistant to lateral transfers (thereby remaining for a longer evolutionary period) [93] while allowing for adaptation and survival in an unstable environment via the plastic portions of the genome [40].

Of the strain-specific genes identified in metatranscriptomic data, those found in strain LSC13-02 were most represented followed by strain LE013-01 and then strain LE3, though it should be noted that the metatranscriptomic data represent multiple *Microcystis* genotypes [24]. While the replication of our analyses was not sufficient to assess its statistical significance, the strain-specific genes in the metatranscriptomic data indicated a shift from coenzyme transport & metabolism, and replication, recombination, & repair early in the bloom to translation, ribosomal structure & biogenesis, signal transduction defense mechanisms, and prophages & transposons in the late bloom. The repeatability and physiological significance of this shift (if any) remain to be investigated, but it could reflect shifts in *Microcystis* population structure within blooms [13], for example between strains being better adapted to withstand either top-down [47] or bottom-up [40, 87] stressors.

The three LGL *Microcystis* strains had different genetic capacities for defense against foreign DNA by using restriction-modification and CRISPR-Cas systems (Table 2, Fig 2), similar to previous findings [36, 80, 92]. Strain LE3 has the most diversity in CRISPR-Cas Subtypes, the most DNA restriction-modification enzymes and the fewest strain-specific genes while strain LSC13-02 had the greatest number of restriction enzymes, a strain-specific restriction-modification protein, less CRISPR-Cas diversity, and the most strain-specific genes. CRISPR-Cas systems are a biochemical mechanism for regulation of genetic exchange in a host, but can also serve a role in determining population structure by changing host-phage dynamics within a bloom [36, 94]. Evidence of viral infection was found in the genomes of strains LSC13-02 and LE013-01, which contained DNA-binding prophage proteins and phage integrases, and phage tail sheath proteins, respectively. Viruses can be a significant top-down selective pressure that can drive microdiversity within an environment even at the earliest stages of divergence, yielding a greater number of subpopulations [13, 94]. Resistance to viral infection through a diversity of CRISPR-Cas systems can sustain subpopulation diversity and overall population stability [95]. This is relevant to our study as *Microcystis* spp. often dominate the bloom biomass in western Lake Erie from mid-July through October [41]. Furthermore, previous studies have found that the dominance of different *Microcystis* strains changes both spatially and temporally during the course of the bloom [82, 96–100].

In conclusion, our results demonstrate that strain-specific genes observed in the newly sequenced genomes of cultured strains of LGL *Microcystis* strains are also present and expressed in high abundance in bloom communities from these same bodies of water. The high degree of genomic plasticity in *Microcystis* has been attributed to a strategy for adapting to variable environments, such as those found in many freshwater ecosystems [31]. Our work contributes to the available data of *Microcystis* strains from the LGL and our capacity to interpret the ecological relevance of metagenomic and metatranscriptomic data [101]. Based on our data on the abundance and expression of the flexible genome in natural communities and its phylogenetic link to the core genome we hypothesize that differences in the flexible portion of the *Microcystis* genome encodes ecologically relevant variation between strains. The nature of these phenotypes, and whether they qualify as ecotypes, remains to be determined. However, previous studies suggest that key traits that vary between strains are toxicity, competition for light, and capability for uptake of nitrogen and carbon, and accordingly that shifts in the strain composition of *Microcystis* blooms are associated with changes in the availability of light, nitrogen, and/or carbon [33, 47, 87]. Blooms are typically associated with many microbial antagonists that are potential top-down controls on *Microcystis* [102]. Our results are consistent with a variable genomic capacity to resist foreign genetic elements and represents an adaptive strategy for *Microcystis* in freshwater ecosystems. Such differences between strains would lead to population re-structuring when exposed to either top-down or bottom-up controls and may be a driver of bloom composition throughout bloom events.

## Supporting information

S1 Fig Estimates of the core and pan-genome of *Microcystis* based on 3 newly sequenced genomes and 17 publicly available genomes (obtained from IMG). A- Core-genome based on exponential decay models of Tetteline et al. (Blue) and Willenbrock et al. (Red) fitted to ten random resamplings of OMCL core-genome clusters. B- Estimate of the pan-genome based on the model of Tettelin et al. fitted to ten random resamplings of OMCL gene clusters.
(DOCX)

S1 Text Comparative genomics pipeline and example commands and scripts. For additional scripts check https://github.com/Geo-omics, or contact the corresponding author.
(DOCX)
