# Supplementary material for: Genome sequences of lower Great Lakes Microcystis sp. reveal strain-specific genes that are present and expressed in western Lake Erie blooms
Source: PLoS One. 2017 Oct 11;12(10):e0183859. doi: 10.1371/journal.pone.0183859 (PMC5647855; doi:10.1371/journal.pone.0183859)

**S1 Fig**

Estimates of the core and pan-genome of *Microcystis* based on 3 newly sequenced genomes and 17 publicly available genomes (obtained from IMG). A- Core-genome based on exponential decay models of Tettelin et al. (Blue) and Willenbrock et al. (Red) fitted to ten random resamplings of OMCL core-genome clusters. B- Estimate of the pan-genome based on the model of Tettelin et al. fitted to ten random resamplings of OMCL gene clusters.


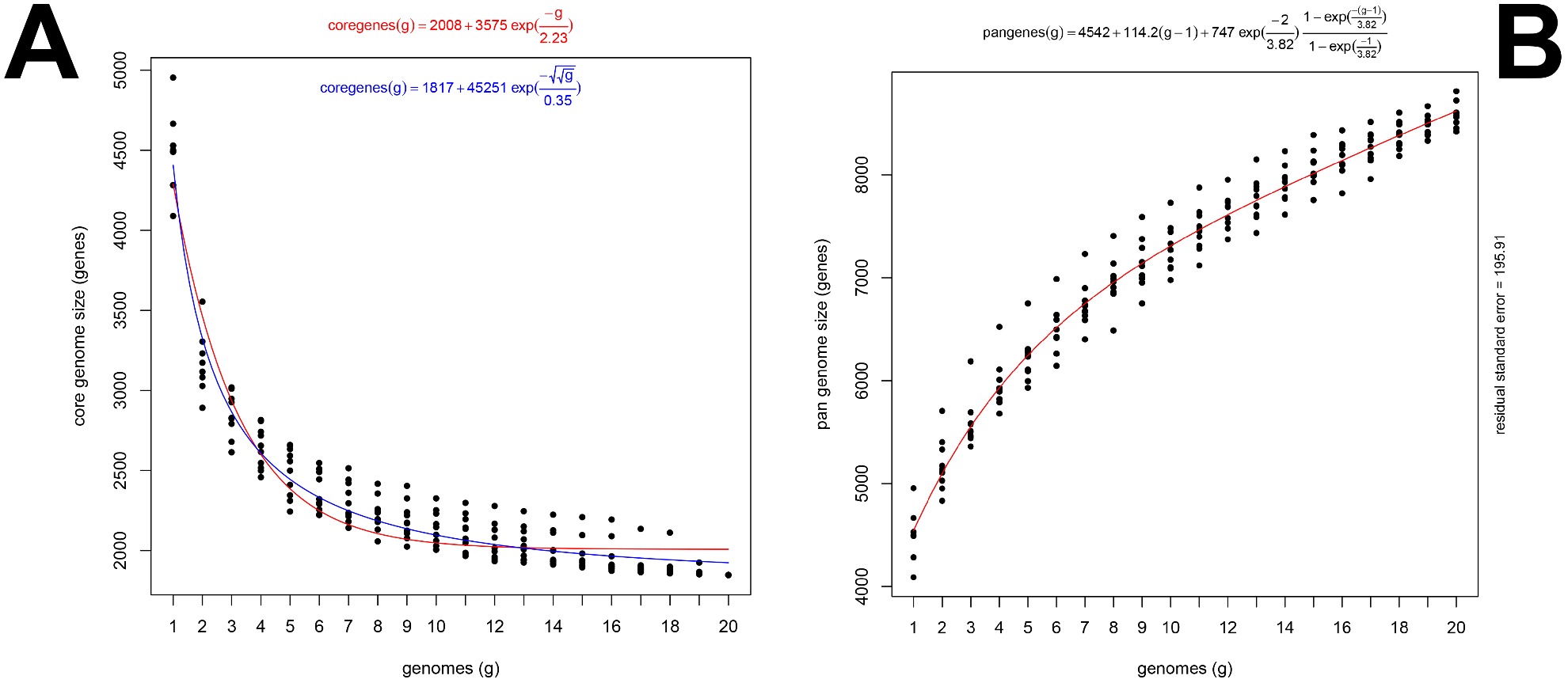

Supplement: S1 Fig — A- Core-genome based on exponential decay models of Tetteline et al. (Blue) and Willenbrock et al. (Red) fitted to ten random resamplings of OMCL core-genome clusters. B- Estimate of the pan-genome based on the model of Tettelin et al. fitted to ten random resamplings of OMCL gene clusters. (DOCX) [file pone.0183859.s001.docx]
