# Supplementary material for: Genome sequences of lower Great Lakes Microcystis sp. reveal strain-specific genes that are present and expressed in western Lake Erie blooms
Source: PLoS One. 2017 Oct 11;12(10):e0183859. doi: 10.1371/journal.pone.0183859 (PMC5647855; doi:10.1371/journal.pone.0183859)
Supplement: S1 Text — For additional scripts check https://github.com/Geo-omics, or contact the corresponding author. (DOCX) [file pone.0183859.s002.docx]

**S1 Text**

Comparative genomics pipeline and example commands and scripts. For additional scripts check <https://github.com/Geo-omics>, or contact the corresponding author.

####PRODIGAL FORMATTING####

#Download Genbank files of publically available genomes of interest

#Extract nucleic acid sequences from Genbank files using gbk2fna.pl

for i in ../GBK/*.gbk; do myGbk=${i##*/}; myFNA=${myGbk%.*}.fna; echo -e "$i\t$myFNA"; perl gbk2fna.pl -in $i -out $myFNA & done

#Add IMG *.fna files for new genomes to the same directory

####RUNNING PRODIGAL####

#We chose Microcystis aeruginosa NIES-843 as a training genome due to it being a complete (Closed) genome

Prodigal -i Microcystis_aeruginosa_NIES_843.fasta -t nies843.trn

#Create *.gbk *.faa *.fna and *.score files for all genomes via Prodigal, putting each in a separate directory

for i in FNA/*.fna; do myFile=${i##*/}; myFileName=${myFile%.*}; echo -e "$i\t$myFileName"; mkdir -p prodigal_{gbk,aa,nuc,score}; prodigal -i $i -o prodigal_gbk/${myFileName}.gbk -a prodigal_aa/${myFileName}.faa -d prodigal_nuc/${myFileName}.fna -s prodigal_score/${myFileName}.score -t nies843.trn &> prodigal_${myFileName}.log & done

#Move log files into a new directory

Mkdir prodigal_log

Mv *.log prodigal_log

#Rename headers in prodigal output for Get_homologues

#From within the Prodigal folder

#Get alias.pl and run

#Move output into Aliased folder

#From within Aliased folder

mkdir RENAMED

for i in *.faa; do myName=${i%%.*}; mySuffix=$(echo $myName | sed "s#_# #"); echo $mySuffix; perl renameHeaders.pl -f $i -suffix "[${mySuffix}]" -d " " -o RENAMED/${myName}.faa; done

for i in *.fna; do myName=${i%%.*}; mySuffix=$(echo $myName | sed "s#_# #"); echo $mySuffix; perl renameHeaders.pl -f $i -suffix "[${mySuffix}]" -d " " -o RENAMED/${myName}.fna; done

#GET_HOMOLOGUES analysis, following the process outlined in the Get_Homologues manual Contreras-Moreira and Vinuesa, 2014

####STEP 1####

#LOAD get_homologues and R

module load get_homologues/1.4

module load R/3.2.1

mkdir Workflow_logs

mkdir Renamed_prodigal_files

cd Renamed_prodigal_files

ln -s ../../Prodigal/Aliased/RENAMED/*.faa .

ln -s ../../Prodigal/Aliased/RENAMED/*.fna .

####STEP 2####

#Calculate blast results and produce BDBH clusters

nohup get_homologues.pl -d Renamed_prodigal_files/ -c -n 30 &> Workflow_logs/1_BDBHclusters.log &

#Produce COGx clusters and report core & pan genome

nohup get_homologues.pl -d Renamed_prodigal_files/ -G -c -n 30 -t 0 -x &> Workflow_logs/1_COGxclusters.log &

#Produce OMCL clusters and report core & pan genome

nohup get_homologues.pl -d Renamed_prodigal_files/ -M -c -n 30 -t 0 &> Workflow_logs/1_OMCLclusters.log &

#Go into cluster directories and determine the number of clusters

ls | wc

####STEP 3####Consensus Core & Pan Genome

#Generate a consensus core genome with all algorithms

nohup compare_clusters.pl -o Intersections_core -d Renamed_prodigal_files_homologues/Microcystiswesenbergii39862LE013-01Bin42_f0_alltaxa_algBDBH_e0_/,Renamed_prodigal_files_homologues/Microcystiswesenbergii39862LE013-01Bin42_f0_0taxa_algCOGx_e0_/,Renamed_prodigal_files_homologues/Microcystiswesenbergii39862LE013-01Bin42_f0_0taxa_algOMCL_e0_/ -m -t 20 &> Workflow_logs/2-1_Compare_core_BDBH-COGx-OMCL.log &

cd Intersections_core

mkdir All_algorithms

mv *.faa intersection_t20.cluster_list pangenome_matrix_t20.* unique_Microcystiswesenbergii39862LE013-01Bin42_f0_* venn_t20.pdf All_algorithms/

#Generate a consensus core with COGx & OMCL (algorithms used in the pan genome calculations)

nohup compare_clusters.pl -o Intersections_core/ -d Renamed_prodigal_files_homologues/Microcystiswesenbergii39862LE013-01Bin42_f0_0taxa_algCOGx_e0_/,Renamed_prodigal_files_homologues/Microcystiswesenbergii39862LE013-01Bin42_f0_0taxa_algOMCL_e0_/ -m -t 20 &> Workflow_logs/2-2_Compare_core_COGx-OMCL.log &

cd Intersections_core

mkdir COGx-OMCL

mv *.faa intersection_t20.cluster_list pangenome_matrix_t20.* unique_Microcystiswesenbergii39862LE013-01Bin42_f0_0taxa_alg* venn_t20.pdf COGx-OMCL/

#Generage a consensus pan genome (COGx & OMCL combined)

nohup compare_clusters.pl -o Intersections_pan -d Renamed_prodigal_files_homologues/Microcystiswesenbergii39862LE013-01Bin42_f0_0taxa_algCOGx_e0_/,Renamed_prodigal_files_homologues/Microcystiswesenbergii39862LE013-01Bin42_f0_0taxa_algOMCL_e0_/ -t 0 -m -T &> Workflow_logs/3_Compare_pan_COGx-OMCL.log &

####STEP 4####Individual Core & Pan Genomes

#Calculate Core genomes for COGx & OMCL (From within the *_homologues directory)

nohup plot_pancore_matrix.pl -i core_genome_algCOGx.tab -f core_both&> ../Workflow_logs/4-1_Calc_core_COGx.log &

nohup plot_pancore_matrix.pl -i core_genome_algOMCL.tab -f core_both&> ../Workflow_logs/4-2_Calc_core_OMCL.log &

#Calculate Pan genomes

nohup compare_clusters.pl -o Intersections_pan/COGx/ -d Renamed_prodigal_files_homologues/Microcystiswesenbergii39862LE013-01Bin42_f0_0taxa_algCOGx_e0_ -t 0 -m -T &> Workflow_logs/4-3_Pan_COGx.log &

nohup plot_pancore_matrix.pl -i pan_genome_algCOGx.tab -f pan &>../Workflow_logs/4-4_Calc_pan_COGx.log

nohup compare_clusters.pl -o Intersections_pan/OMCL/ -d Renamed_prodigal_files_homologues/Microcystiswesenbergii39862LE013-01Bin42_f0_0taxa_algOMCL_e0_ -t 0 -m -T &> Workflow_logs/4-5_Pan_OMCL.log &

nohup plot_pancore_matrix.pl -i pan_genome_algOMCL.tab -f pan &> ../Workflow_logs/4-6_Calc_pan_OMCL.log &

#Compare *.tab_core_both.log files to find best residual Standard error

#Tettelin fit rSE: COGx 167.78, OMCL 173.75

#Willenbrock fit rSE: COGx 128.60, OMCL 133.47

#Core genome size (Based on Willenbrock fit): COGx 1978.6, OMCL 1924

#Compare *_Pfam.tab_pan.log files to find best residual Standard error

#rSE: COGx 193.84, OMCL 195.91

#Pan-genome size: COGx 8259.6, OMCL 8619.8

####STEP 5####Classify genes

#Must be in directory containing relevant pangenome_matrix_t0.tab file

nohup parse_pangenome_matrix.pl -m pangenome_matrix_t0.tab -s &>../Workflow_logs/5-1_Classify_genes_COGx-OMCL.log

nohup parse_pangenome_matrix.pl -m pangenome_matrix_t0.tab -s &> ../../Workflow_logs/5-2_Classify_genes_COGx.log &

nohup parse_pangenome_matrix.pl -m pangenome_matrix_t0.tab -s &> ../../Workflow_logs/5-3_Classify_genes_OMCL.log &

####STEP 6####Create .txt lists to parse unique genes, with two lists for each comparison (genomes in include-those you’re interested in and genomes to exclude)

####STEP 7####Find unique genes using parse function (From within directory of your choosing with pangenome_matrix_t0.tab file

#NOTE: the -p function will yield an error for plotting function (input not from/in .gbk) but WILL successfully parse and name output file according to -p flag

#Example script

parse_pangenome_matrix.pl -A ../GrLks_vs_Other_A.txt -B ../GrLks_vs_Other_B.txt -g -m pangenome_matrix_t0.tab -p _GrLks_vs_other

####STEP 8####BLAST prep

#For parse results with multiple hits (list of .faa files) you must concatenate the results into a single file that can be BLASTed. To do so, open the *_pangenes_list files in Notepad++, convert to Unix/OSX (Edit>EOL Conversion>Unix/OSX), then make the list into a single line (Edit>Line Operations>Join Lines), copy this line into the cat command from within the directory of the parse command. Cat "List" > "Comparison"_faa_list.faa

mkdir BLAST

cd BLAST

#Load current BLAST module

#Create databases using annotated *.faa files

#NIES-843 (Reference) database

cp ../../Prodigal/GBK/FAA/Microcystis_aeruginosa_NIES_843.faa .

makeblastdb -in Microcystis_aeruginosa_NIES_843.faa -dbtype prot

#All-genomes database

cat ../../Prodigal/GBK/FAA/*.faa ../../Prodigal/prodigal_aa/Microcystis_aeruginosa_NIES_44.faa /geomicro/data21/HABs/Run_1105/IMG/Microcystis_Bins/Microcystis_aeruginosa_39870_LE3_Bin_41/2606217223.genes.faa /geomicro/data21/HABs/Run_1105/IMG/Microcystis_Bins/Microcystis_cf_aeruginosa-botrys_39860_LSC13-02_Bin_42/2606217221.genes.faa /geomicro/data21/HABs/Run_1105/IMG/Microcystis_Bins/Microcystis_wesenbergii_39862_LE013-01_Bin_42/2606217222.genes.faa > Micro_blast_db.faa

makeblastdb -in Micro_blast_db.faa -dbtype prot

#Get mapper.pl for mapping of reads

cp /geomicro/data1/COMMON/scripts/BlastTools/mapper.pl .

####STEP 9####COGx unique vs. reference database

nohup blastp -query ../Intersections_pan/COGx/LE3_vs_ALL_faa_list.faa -db Microcystis_aeruginosa_NIES_843.faa -outfmt "6 std qcovs slen stitle" -out COG_LE3_vs_NIESdb.blastp &> ../Workflow_logs/6-1_COGx_LE3_vs_NIES_blastp.log &

nohup blastp -query ../Intersections_pan/COGx/LSC13-02_vs_ALL_faa_list.faa -db Microcystis_aeruginosa_NIES_843.faa -outfmt "6 std qcovs slen stitle" -out COG_LSC13-02_vs_NIESdb.blastp &> ../Workflow_logs/6-2_COGx_LSC_vs_NIES_blastp.log &

nohup blastp -query ../Intersections_pan/COGx/LE013-01_vs_ALL_faa_list.faa -db Microcystis_aeruginosa_NIES_843.faa -outfmt "6 std qcovs slen stitle" -out COG_LE013-01_vs_NIESdb.blastp &> ../Workflow_logs/6-3_COGx_LE013-01_vs_NIES_blastp.log &

####Here is a good spot to use postBlast filtering to remove anything with a query and subject coverage <75 (get_homologues standard)

#From within the directory containing Blast output

#Use mapper to find partial genes (for manual clustering)

perl mapper.pl -b COG_LE3_vs_NIESdb.blastp -q ../Intersections_pan/COGx/LE3_vs_ALL_faa_list.faa -o COG_LE3 -p 90 -d 25

perl mapper.pl -b COG_LSC13-02_vs_NIESdb.blastp -q ../Intersections_pan/COGx/LSC13-02_vs_ALL_faa_list.faa -o COG_LSC -p 90 -d 25

perl mapper.pl -b COG_LE013-01_vs_NIESdb.blastp -q ../Intersections_pan/COGx/LE013-01_vs_ALL_faa_list.faa -o COG_LE013 -p 90 -d 25

#Manually look at blastp output to make sure there are no "false positive" (clusters get_homologues called as unique that are not actually unique)

##REPEAT for each algorithm of interest

####STEP 10####COG unique vs. whole database

nohup blastp -query ../Intersections_pan/COGx/LE3_vs_ALL_faa_list.faa -db Micro_blast_db.faa -outfmt "6 std qcovs slen stitle" -out COG_LE3_vs_wdb.blastp &> ../Workflow_logs/6-4_COG_LE3_vs_wdb_blastp.log &

nohup blastp -query ../Intersections_pan/COGx/LSC13-02_vs_ALL_faa_list.faa -db Micro_blast_db.faa -outfmt "6 std qcovs slen stitle" -out COG_LSC13-02_vs_wdb.blastp &> ../Workflow_logs/6-5_COG_LSC_vs_wdb_blastp.log &

nohup blastp -query ../Intersections_pan/COGx/LE013-01_vs_ALL_faa_list.faa -db Micro_blast_db.faa -outfmt "6 std qcovs slen stitle" -out COG_LE013-01_vs_wdb.blastp &> ../Workflow_logs/6-6_COG_LE013-01_vs_wdb_blastp.log &

####STEP 11####Mapping metagenome to complete genomes

cd LE3_Mapping

mkdir Sample_42896_vs_LE3_Pre_cluster

ln -s ../../Renamed_prodigal_files/Microcystis_aeruginosa_39870_LE3_Bin_41.fna scaffold.fasta

ln -s /geomicro/data21/HABs/Run_1220/Project_denef/Sample_42896/42896_fwd.fastq fwd.fastq

ln -s /geomicro/data21/HABs/Run_1220/Project_denef/Sample_42896/42896_rev.fastq rev.fastq

nohup bash mapping.sh Sample_42896_vs_LE3_Pre_cluster &> ../Workflow_logs/7-6_42896_vs_LE3_whole_pre_cluster.log &

##REPEAT for each Sample/Strain of interest

####STEP 12####BLAST to annotated genomes to get annotations for proteins/clusters

#See additional README_for_annotations for information

nohup bash Annotation_BLAST.sh &> Workflow_logs/8-1_Annotation_BLAST.log &

#Filter BLAST results using postBlast script which was implemented by another "wrapper" script

nohup bash postBlast_all.sh &> ../Workflow_logs/8-2_PostBLAST_filtering.log &

#Map BLAST results to NCBI/annotated genes using a script

nohup bash batch_mapper.sh &> 8-3_BLAST_mapping.log &

####STEP 13####Get annotations using BLAST and mapping results using spreadsheet/lookup functions with BLAST outputs, mapping outputs, and coverage files

####STEP 14####Assessing metagenomic and metatranscriptomic coverage of both unique genes and housekeeping genes (hypothetical/new Figure 4)

#From within /omics/HABs/Microcystis/GH_Prodigal_20gnms/*STRAIN*_Mapping directories

#Make links to the appropriate scaffold and read files in each directory

#Scaffold files are for the genome you want coverage data on, in this case the "completed" Microcystis genomes LE3, LSC13-02, and LE013-01 (Look for IMG output/data for a GENOME)

#Read files are for the metagenome or metatranscriptome you want to map onto your scaffolds (to see what they cover and how much) NOTE: this should be two files (fwd and rev) for metagenome and ONE file for metatranscriptome

#Example Strain LE3 (mapping reads TO strain LE3)

ln -s /geomicro/data21/HABs/Run_1105/Sample_39870/Assembly/k52_92_s8/scaffold.fa scaffold.fasta####

##REMINDER: mapping.sh for metagenomes (paired-end) but mapping_singles.sh for transcriptomes (single-end)

#Get mapping scripts and load dependencies

cp /geomicro/data1/COMMON/scripts/BashScripts/mapping.sh .

cp /omics/HABs/Microcystis/GH_Prodigal_20gnms/LE3_Mapping/mapping_singles.sh .

ln -s /geomicro/data1/COMMON/scripts/BamTools/coveragePerScaffold.pl .

module load bedtools/2.23.0 bwa/0.7.9a samtools/1.0.0

nohup bash mapping.sh Sample_42895 &> ../Workflow_logs/15-1_42895_Map2_IMG_LE3.log &

####Step 15####

#Using bedtools to get gene-by-gene coverage of Microcystis genomes to assess coverage of unique & housekeeping genes (For Figures 4&5 in manuscript)

####NOTE####

#Need to create .bed files for all prodigal-predicted genes consisting of 3 columns; scaffold name (whole scaffold), gene start, gene stop and use this

#Example For LE3

nohup bedtools multicov -bams ../Sample_42895/scaffold_sorted.bam ../Sample_42896/scaffold_sorted.bam ../Transcript_50632/scaffold_sorted.bam ../Transcript_53185/scaffold_sorted.bam ../Transcript_53187/scaffold_sorted.bam ../Transcript_53189/scaffold_sorted.bam ../Transcript_53191/scaffold_sorted.bam ../Transcript_53193/scaffold_sorted.bam ../Transcript_53195/scaffold_sorted.bam -bed /omics/HABs/Prodigal/Microcystis_aeruginosa_39870_LE3_Bin_41.bed &> LE3_prodigal_genecov.out &

#To intrepret the output you need the following

#Housekeeping gene list

#Unique gene list (W/annotations)

#Prodigal Aliased AKA file

#IMG .gff file used in the multicov step

#Using RH-script generated .gff copy locus tags to *_prodigal_genecov.out

#Using prodigal .aka file, VLOOKUP locus tags to get Prod_ID (OR just start at top with M0000000001 and fill to bottom)

#Make new tab/sheet (HK9_Genes): populate this with the HK9: Scaffold, start, stop, GeneID, locus tag, product, MG&MG coverage ####NOTE: double check start/stop to get correct gene (some locus tags will differ)

#NEW tab/sheet (Unique_genes): populate this with: Prod_ID, scaffold, start, stop, gene_ID, unq_gene_SA, Prodigal_locus_tag, product, MG&MT coverage

####Step 16####use BLAST to 1- double check mapping data for unique genes and 2- get read/coverage info for unique genes

#Make sure BLAST modules are loaded

module load blast/2.2.30+

#Create the files for the database (Fasta files of unique genes)

#Using Excel and Notepad++ open the pangenome_matrix_t0__*_vs_ALL_pangenes_list.txt files for each strain, isolate just the gene "name" (should be a M followed by 10 digits), put these into a single line with names separated by a "Bar" |

#From within Renamed_prodigal_fiels directory grep for the unique gene names and following line (nucleotide sequence)

#Make appropriate files for BLASTS (Example for LE3)

#Amino acid file

ln -s ../../Intersections_pan/LE3_vs_ALL_faa_list.faa LE3_vs_ALL_unique.fasta

#Recently created nucleic acid file

mv ../../Renamed_prodigal_files/LE3_Uniques.fasta .

#Metagenome assembly

ln -s /geomicro/data21/HABs/Run_1220/Project_denef/Sample_42895/Assembly/k52_92_s8/scaffold.fa 42895_assembled.fasta

ln -s /geomicro/data21/HABs/Run_1220/Project_denef/Sample_42896/Assembly/k52_92_s8/scaffold.fa 42896_assembled.fasta

#Metatranscriptome examples

ln -s /omics/HABs/Microcystis/Transcripts/Sample_50632/50632_fwd.fasta 50632_fwd.fasta

makeblastdb -in LE3_Uniques.fasta -dbtype nucl

nohup blastn -query 42895_assembled.fasta -db LE3_Uniques.fasta -outfmt "6 std qcovs stitle" -out LE3_vs_42895.blastn -num_threads 10 &>../../Workflow_logs/17-1_LE3_uniques_vs_42895_BLAST.log &

#REPEAT for all strains

##############################################################################
